# Transient Oculomotor Palsy after Influenza Vaccination: Short Report

**DOI:** 10.5402/2011/849757

**Published:** 2011-04-26

**Authors:** Diogo Fraxino de Almeida, Adilson Teixeira Teodoro, Rafael de Figueiredo Radaeli

**Affiliations:** ^1^Department of Medicine, Universidade Estadual de Maringá (UEM), Av. Mandacaru 1590, Maringá, 87083-240, PR, Brazil; ^2^Eletroneuro Clínica Médica, Av. Independência 258, Sala 405, Maringá, 87015-020, PR, Brazil; ^3^Unimed Bebedouro Hospital, Rua Marechal Floriano Peixoto 713, Bebedouro, 14701-360, SP, Brazil

## Abstract

Several neurological complications have been described after influenza vaccination. Oculomotor palsy has not been yet related with influenza vaccine. We report a 79-year-old man who developed an acute transient right oculomotor palsy two days after a routine influenza vaccination, for which no other cause was identified. There was no evidence of diabetes, glucose intolerance, high blood pressure, hipercholesterolemia, smoking, obesity, systemic vasculitis, or other risk factor for ischemic neuropathy. The cerebrospinal fluid was normal as well as the MRI and MRA scans. The differential diagnosis and the possible relationship between the vaccine and the oculomotor palsy are discussed.

## 1. Introduction

The influenza vaccine is considered safe and is widely recommended to prevent influenza virus infection complications in high risk populations [1]. Side effects include soreness at the vaccination site, fever, malaise, and myalgias, especially in patients who had never exposed to the influenza virus antigens present in the vaccine [2, 3]. 

Neurological complications have been described after influenza vaccination, including Guillain-Barre syndrome [4–8], chronic inflammatory demyelinating polyneuropathy (CIDP) [9], acute disseminated encephalomyelitis [10–12] acute transverse myelitis [12–15], optic neuritis [16, 17], cerebellar ataxia [18], giant cell arteritis [19], dermatomyositis [20], hypoglossal palsy [21], peripheral facial palsy [22–24] and vasculitic ulnar mononeuropathy [25]. As far as we know oculomotor mononeuropathy has not been described as a complication of influenza vaccine. We report a patient with sudden development of painful right oculomotor palsy following influenza vaccination with complete recovery after two weeks.

## 2. Case Report

A 79-year-old white male presented with a history of sudden development of right painful ophthalmoplegia, diplopia, and palpebral ptosis accompanied by nausea, vomiting, and headache. The symptoms developed two days after the influenza vaccination (Vaxi-grip, Aventis Pasteur, Lyon, France). The vaccine contained inactivated A/New Caledonia/20/99(H1N1)-like virus, A/Wisconsin/67/2005(H3N2)-like virus, B/Malaysia/2506/2004-like virus, which had been recommended for the 2007 season on the South hemisphere. There was no history of diabetes, glucose intolerance, arterial hypertension, hypercholesterolemia, systemic vasculitis, smoking, obesity, or other risk factors for ischemic oculomotor nerve palsy. There was no personal or family history of neurological disorders.

General physical examination was normal. The blood pressure was 130/80 mmHg. The neurological examination showed right oculomotor palsy with pupil preservation. All other cranial nerves were preserved. The muscle strength, deep tendon reflexes, and muscle tone and coordination were normal. The plantar response was normal bilaterally. Sensory examination did not show abnormalities. The gait was normal, and Romberg test was negative.

The workup showed normal red and white blood cell count as well as platelet count, sedimentation rate, sodium, potassium, calcium, urea, creatinine, total cholesterol, triglycerides, fast glucose, glucose tolerance test, and glycosylated hemoglobin levels. The routine cerebrospinal fluid was normal. The head CT scan did not show any abnormality as well as brain magnetic resonance imaging (MRI) and magnetic resonance angiography (MRA). All tests were done following informed consent of the patient, who agreed with the publication of the case.

The pain was treated with symptomatic analgesics. After two weeks of symptoms, a gradual and complete recovery was observed, without any specific treatment.

## 3. Discussion

Neurological complications are rare after influenza vaccination, usually restricted to a few case reports [25]. The exceptions were the Guillain-Barre syndrome outbreak in the USA in 1976 and 1977 after a massive use of the swine-flu vaccine in over than 40 million people and the peripheral facial palsy outbreak in Switzerland between 2000 and 2001 after the use of the intranasal presentation of the vaccine, both no longer used nowadays [7, 24]. Most of the cases occurred within 4 weeks after the vaccination. The current inactivated influenza vaccine is considered safe [26]. In 1990, the Vaccine Adverse Events Reporting System (VAERS) was criated by the Centers for Disease Control for vigillance of vaccine side effects. Lasky et al. [6] have found an incidence of 1-2 Guillain-Barre cases/1.000.000 of vaccinated between 1992 and 1994. Others reports have found a small increased risk of GBS after influenza vaccination in the subsequent years [4, 27]. 

Sudden oculomotor palsy with or without other neurological deficits can occur in several different conditions along the pathway of the nerve from the brainstem to the extrinsic eye muscles inside the orbit. Midbrain, interpeduncular cistern, cavernous sinus and orbit are some of the sites of possible injury of the oculomotor nerve through its pathway. The oculomotor palsy can be complete when the pupil is affected and incomplete when it is spared. Usually incomplete lesions are caused by ischemia of the nerve in patients with diabetes, arterial hypertension, obesity, smoking, or a combination of risk factors [28–30]. On the other hand, complete lesions are most frequently observed in compressive lesions of the nerve such as aneurysm of the posterior communicating artery, since the parasympathetic fibers are localized in the periphery of the nerve. 

Since all possible underlying structural causes of oculomotor nerve injury were ruled out by clinical, laboratorial, and imaging workup, the influenza vaccine may be the causal factor of the transient oculomotor nerve palsy in the present case. The influenza vaccine probably causes damage to the myelin sheaths and surrounding axons in complications such as Guillain-Barre syndrome, acute disseminated encephalomyelitis, transverse myelitis, Bell's palsy, and optic neuritis. In theory, it is probable that it could trigger oculomotor palsy by the same mechanism. The fast recovery of the symptoms in our patient corroborates the hypotesis of demyelination, althought there is no pathological comprovation for this theory. 

 Although it has not been possible to establish a definite causal relationship between the influenza vaccine and the oculomotor palsy in the present case, no better explanation was found. It is widely known that ischemia is usually the cause of incomplete oculomotor palsy; however, the absence of recognized risk factors for atherosclerosis in our patient, except for advanced age, makes this possibility unlikely. Thus, we would like raising the possibility of such complication. Since vaccines may cause central and peripheral nervous system disorders, we think being reasonable to consider this possibility at the differential diagnosis. This is the first oculomotor palsy possibly associated with influenza vaccination described in the literature to date. A case of isolated oculomotor palsy has been previously reported after measles vaccination in a child [31]. Further population based studies are necessary to prove the relationship between influenza vaccine and oculomotor palsy, as well as to find out its underlying mechanisms.

Although influenza vaccine is routinely recommended in high-risk groups, rare neurological complications, including oculomotor palsy can be seen. However, it is important to highlight that such complications do not outweigh the beneficial effects of the influenza vaccine in high risk populations [32].
